# Developmental assistance for child and adolescent mental health in low– and middle–income countries (2007–2014): Annual trends and allocation by sector, project type, donors and recipients

**DOI:** 10.7189/07.020901

**Published:** 2017-12

**Authors:** Jasmine Turner, Hugo Pigott, Mark Tomlinson, Mark JD Jordans

**Affiliations:** 1 Department of Research and Development, War Child Holland, the Netherlands; 2 Independent consultant, Accra, Ghana; 3 Department of Psychology, Stellenbosch University, Stellenbosch, South Africa; 4 Centre for Global Mental Health, London School of Hygiene and Tropical Medicine, London, UK

## Abstract

**Background:**

Globally, mental disorders are the leading cause of disability among children and adolescents. To date, there has been no estimate of developmental assistance supporting mental health projects that target children and adolescents (DAMH–CA). This study aimed to identify, describe and analyse DAMH–CA with respect to annual trends (2007–2014), sector, project type, recipient regions, and top donor and recipient countries, and estimate annual DAMH–CA per child/adolescent by region.

**Methods:**

Developmental assistance for all projects focused on children and adolescent mental health between 2007 and 2014 was identified on the Organisation for Economic Co–operation and Development’s (OECD) Creditor Reporting System, and analysed by target population, sector, project type, donors, and recipients. The study did not include governmental or private organisation funds, nor funding for projects that targeted the community or those that included mental health but not as a primary objective.

**Results:**

Between 2007 and 2014, 704 projects were identified, constituting US$ 88.35 million in DAMH–CA, with an average of 16.9% of annual development assistance for mental health. Three quarters of DAMH–CA was used to fund projects in the humanitarian sector, while less than 10% was directed at mental health projects within the education, HIV/AIDS, rights, and neurology sectors. DAMH–CA was predominantly invested in psychosocial support projects (US$ 63.24 million, 72%), while little in absolute and relative terms supported capacity building, prevention, promotion or research, with the latter receiving just US$ 1.2 million over the eight years (1.4% of total DAMH–CA). For 2014, DAMH–CA per child/adolescent was US$ 0.02 in Europe, less than US$ 0.01 in Asia, Africa, and Latin America and the Caribbean, and US$ 0 in Oceania.

**Conclusions:**

To mitigate the growing burden of mental and neurological disorders, increased financial aid must be invested in child and adolescent mental health, especially with respect to capacity building, research and prevention of mental disorder projects. The present findings can be used to inform policy development and guide resource allocation, as current developmental assistance is described by sector and project type, thereby facilitating the identification of specific areas of investment need.

The Global Burden of Disease Study 2013 indicates that 21.2% of years lost to disability (YLDs) and 7.1% of disability–adjusted life years (DALYs) are attributable to mental illness, although a more recent analysis suggests that 32.4% and 13.0%, respectively, are more accurate estimates due to exclusion/inclusion issues with the original calculations [1,2]. An estimated 20% of all children and adolescents have some form of mental disorder, one quarter of which are severe [3,4]. Globally, mental illness (including substance use disorders) is the leading cause of disability in children and young people, accounting for 54.2 million YLDs (25% of disability in children and youth), 6.3 million years of life lost (YLL) due to suicide, and 61.8 million DALYs (5th leading cause of disability and 6.3% of all DALYs, when including the burden of suicide) [4]. Despite this, the vast majority of children around the world do not have access to mental health services, largely due to severe shortages of mental health professionals, lack of health worker training on children and adolescent mental health, and stigma [5–8]. Given that an estimated 50% of adult mental disorders have their onset during adolescence, the lack of care and early intervention during this period results in lost economic productivity by both the individual and their carers [8–10]. The World Health Organisation (WHO), researchers, advocates and clinicians repeatedly recommend prioritising increased prevention of mental disorders, and the adoption of a life–course approach to address the mental health burden [11–13].

Significant strides have been made in recent years to increase the visibility and funding of mental health. The WHO has published a comprehensive mental health action plan (2013–2020) and, with the World Bank, mobilised a global alliance to scale up implementation and prioritise mental health [14–16]. Additionally, mental health and substance use disorders have been included in two health targets of the Sustainable Development Goals [17]. Despite this progress, there is a need for increased funding to support the growth of mental health services. In 2007, the Lancet Global Mental Health Group recommended an investment of US$ 2 per person per year to scale up basic mental health care packages in LICs, and US$ 3–4 in LMICs which, when taking inflation into account, is equivalent to US$ 2.34 and US$ 3.51–4.68 in 2017 US dollars [18,19]. In practice, average governmental spending on mental health ranges from 0.5% (equivalent to US$ 0.20 per capita) of the annual health budget in low–income countries (LICs), 1.9% in low–middle–income countries (LMICs), and 2.4% and 5.1% in upper–middle– and higher–income countries (UMCs and HICs, respectively) [20].

A recent analysis of developmental assistance for mental health (DAMH) between 2007 and 2013 reveals a similar picture, with assistance more than trebling in actual terms from US$ 53.67 million in 2007 to US$ 196.62 million in 2013, yet remaining less than 1% of total developmental assistance for health [21]. The annual DAMH average of US$ 133.57 million is over 50 times less than funding for HIV, despite mental, neurological and substance use disorders causing more than double the global burden of HIV/AIDS [22]. DAMH per capita was calculated to be US$ 0.05 which, when added the average governmental spending, still leaves each individual in LICs US$ 2.09 short per year (accounting for inflation) of the minimum investment needed to scale up basic mental health care packages [21].

To the best of our knowledge, to date there is no estimate of DAMH specifically targeted at children and adolescents (DAMH–CA). The present study aims to estimate DAMH–CA between 2007 and 2014, and to describe, analyse and comment on the distribution by year, sector, project type, top recipients and donors, recipient regions, as well as estimate annual DAMH–CA per child/adolescent by region.

## METHODS

An adaptation of previous studies’ methods using the Creditor Reporting System (CRS) of the Organisation for Economic Co–operation and Development’s (OECD) Development Assistance Committee (DAC) was used to identify DAMH–CA [21,23–25].

### Data source

The CRS is a comprehensive, open–access database detailing aid activities reported by DAC and non–DAC countries, multilateral organisations, and private donors [26]. It provides project–by–project information on donor disbursements (recorded by funding year), and is considered the most reliable source on development assistance projects. CRS spreadsheets detailing all aid projects between 2007 and 2014 were downloaded and searched within for children and adolescent mental health projects (project identification methodology detailed below). Data collected was conducted between November 2015 and May 2016.

### Identification of children and adolescent mental health projects

DAMH–CA is defined here as financial aid for mental health projects targeted at children and/or adolescents. Projects were identified using keywords searches in the project title, short description and long description. The keywords were based on language used for diagnosing and describing mental illness and psychosocial well-being, developed in English and translated into Italian, French, Portuguese, Spanish, German and Dutch (see Table S1 in the **Online Supplementary Document(Online Supplementary Document)**). When a project included a keyword, the title and descriptions were read to determine whether it should be included, and to ascertain and record the target population (community, adults, or children and adolescents). Most of these keywords were the same as those used by previous studies [21] with some additions and/or modifications, eg, “therap–“ to include therapy, therapist, therapeutic. “Wellbeing” was another addition and did present challenges due to the broad concept it encompasses; the project was included if mental well-being was specified, otherwise it was coded as a ‘multicomponent’ project (ie, a project which included mental health within its remit but not as a primary objective).

In line with previous methodologies [21], projects were searched for in the education, health, government and civil services, other social infrastructure and services, and humanitarian aid sectors. Due to the heterogeneity of multicomponent projects in terms of size and aims, it was not possible to identify the proportion of funding directed at mental health therefore, unlike previous studies [21], developmental assistance for multicomponent projects was not included as DAMH–CA.

### Categorisation by sector, project type and population group

Once data collection was complete, a random selection of projects was open–coded for sector and project type by two researchers [JT, HP] (n = 60). The codes were discussed and finalised, and then the remaining projects were manually coded (see Text S2 for definitions of sector and project type, and Text S1 for detailed description of coding process, both in **Online Supplementary Document(Online Supplementary Document)**).

The data were then tracked for trends in DAMH–CA by year, sector, programme type, donor, and recipient. Programmes targeting the general community were not included to prevent overestimation of DAMH–CA, as it became apparent from project descriptions that the overarching focus of community programmes was on adults, and that children and adolescents were generally secondary beneficiaries at best.

The CRS gives three options for the recording of financial data; *actual disbursement*, *money received,* and *commitment*. It was decided to use *actual disbursement* data, as *money received* was rarely recorded and there were often discrepancies between *actual disbursement* and *commitment*. When *actual disbursement* data were not available, *money received* was used instead, and for the remaining projects (n = 73) *commitments,* multiplied by the *actual disbursement*:*commitment* ratio (1:0.816), were used. DAMH–CA is presented in 2013 constant dollars, ie, adjusted for inflation and using the exchange rates of 2013 [27].

### Recipient and donor data, annual DAMH–CA per child/adolescent, and cumulative DAMH–CA by recipient country

Recipient and donor information for most projects was available, therefore the top ten cumulative (ie, aggregated 2007–2014 DAMH–CA) recipients and donors between 2007 and 2014 were identified. DAMH–CA for unspecified recipients (n = 14) amounted to US$ 4.61 million. It was possible to calculate annual DAMH–CA per child/adolescent by region, using UN Department of Economic and Social Affairs population data (see Text S2 in **Online Supplementary Document(Online Supplementary Document)** for detailed methodology). The percentage of cumulative DAMH–CA per recipient country was mapped, although the West Bank and Gaza were excluded as they received a disproportionate percentage (28.0%) of DAMH–CA, which would have rendered the shading of the rest of the map uninformative. DAMH–CA for unspecified recipient counties was not included in the mapping (US$ 5.85 million, 6.6%), and DAMH–CA for unspecified regions (US$ 4.61 million, 5.2%) was not included for the per child/adolescent calculations.

## RESULTS

### DAMH by target population

DAMH between 2007 and 2014 was US$ 550.79 million, of which DAMH–CA was US$ 88.35 million (16.0%). DAMH targeted at adults was US$ 17.74 million (3.2%), and community DAMH was US$ 444.70 million (80.7%). 704 mental health projects targeting children and adolescents were identified, making the average spending per project almost US$ 125 500.

### Annual trends of DAMH–CA

**Figure 1:**
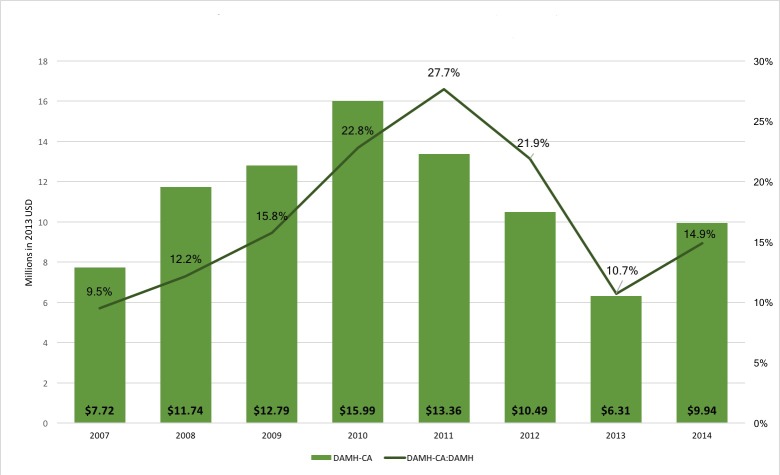
Annual trends of developmental assistance supporting mental health projects that target children and adolescents (DAMH–CA) and as a proportion of DAMH, 2007–2014 (US$ millions).

Annual DAMH–CA between 2007 and 2014 was an average of US$ 11.04 million per year, with no clear annual trend (**Figure 1**). DAMH–CA ranged from a peak in 2010 of US$ 15.99 million to US$ 6.31 million in 2013. DAMH–CA constituted an annual average of 16.9% of DAMH, ranging from 9.5% in 2007 to 27.7% in 2011. Developmental assistance for multicomponent programmes totalled US$ 129.30 million (annual average = US$ 16.16 million). Annually, it varied considerably, from US$ 75.26 million in 2007 to US$ 3.22 million in 2011 (see Figure S1 in **Online Supplementary Document(Online Supplementary Document)**). When considered as a proportion of DAMH, there is possibly a shift from lower to higher annual investment in multicomponent programmes in more recent years, although this would need to be tracked over more years to confirm.

### DAMH–CA by sector and project type

The humanitarian sector received the highest cumulative proportion of DAMH–CA (US$ 41.72 million, 47.2%; **Table 1**). This was followed by the health and substance use sectors (US$ 25.86 million [29.3%] and US$ 10.99 million [12.4%], respectively). Projects addressing autism received US$ 3.91 million over the eight years, equivalent to 4.4% of total DAMH–CA. Other sectors (education, HIV/AIDS, rights, and neurological disorders) received a cumulative total of US$ 5.87 million (6.6% of total DAMH–CA). Many projects supported children and adolescents affected by, or at risk of, specific diseases/disorders, namely substance use, autism, HIV/AIDS and neurological disorders. To provide richer data on the types of projects funded through DAMH–CA, those addressing the mental health impact of these diseases and disorders on children and adolescents have been differentiated from general health projects.

**Table 1:** Distribution of cumulative (2007–2014) DAMH–CA by sector and project type with percentages of sector spending for each project type, US$ millions*

	Capacity building	Prevention	Promotion	Psychosocial support	Research	Sector totals (%)
Humanitarian	0.69 (1.7%)	1.60 (3.8%)	1.76 (4.2%)	37.67 (90.3%)	N/I	**41.72** (47.2%)
Health	3.01 (11.6%)	0.76 (2.9%)	2.18 (8.4%)	19.91 (77.0%)	N/I	**25.86** (29.3%)
Substance use	0.45 (4.1%)	7.17 (65.2%)	0.40 (3.6%)	2.53 (23.0%)	0.44 (4.0%)	**10.99** (12.4%)
Autism	1.74 (44.6%)	N/I	0.18 (4.6%)	1.34 (34.3%)	0.65 (16.6%)	**3.91** (4.4%)
Education	2.10 (61.9%)	N/I	0.65 (19.3%)	0.57 (16.8%)	0.07 (2.0%)	**3.39** (3.8%)
HIV/AIDS	0.20 (16.2%)	0.01 (1.0%)	N/I	1.04 (82.8%)	N/I	**1.26** (1.4%)
Rights	0.05 (7.5%)	N/I	0.64 (92.5%)	N/I	N/I	**0.69** (0.8%)
Neuro	0.32 (60.3%)	N/I	N/I	0.18 (33.3%)	0.03 (6.4%)	**0.53** (0.6%)
Project type totals (%)	**8.57** (9.7%)	**9.54** (10.8%)	**5.81** (6.6%)	**63.24** (71.6%)	**1.20** (1.4%)	**88.35** (100.0%)

DAMH–CA – developmental assistance supporting mental health projects that target children and adolescents, N/I – no investment

*Row percentages show the percentage of DAMH–CA for project types within the different sectors. Percentages in the ‘Sector totals’ column show the percentage that each sector constitutes of the total DAMH–CA.

Over 90% of DAMH–CA in the humanitarian sector was directed at the provision of psychosocial support, where the majority of projects funded counselling and well-being promotion projects. Very little was invested in building the capacity of communities and aid workers to sustain the psychosocial response in the long–term, and none was invested in research. Similarly, the majority of DAMH–CA invested in the general health sector was aimed at the provision of psychosocial support, of which 68% of the projects were funded by UNICEF, predominantly as child friendly spaces. Other project types included training caregivers and counsellors in psychosocial support, well-being promotion projects, and the construction of youth mental health centres. Again, there was no investment in research. Substance use was the only sector which received DAMH–CA for all project types, and which was aimed predominantly at prevention of drug abuse, particularly in South America. Other projects included psychoeducation and improvement of counselling services, and many focused on street children. Much of the DAMH–CA for autism projects funded those supporting and training families to take care of their children’s needs, establishing specialised centres and services, and providing therapy. Over half of the projects in the education sector had a capacity–building focus; the development of mental health services in schools, and construction of specialised centres. Others involved well-being promotion programmes and those supporting cognitive development. There were no projects focusing on prevention of mental disorders within education. Over 80% of DAMH–CA targeting children and adolescents affected directly or indirectly by HIV/AIDS funded the provision of psychosocial support, and most of the remaining assistance was spent on projects that trained counsellors or increased access to counsellors. Very little money – less than 1% of total DAMH–CA – was invested in the promotion of rights of people with mental health problems, but those that were funded frequently supported young people to be advocates for mental health and mental health care. Lastly, most funding within neuro supported the building of observatories, establishment of services, and training of staff for improved care for children and adolescents with neurological and intellectual disabilities.

Capacity building projects frequently focused on building centres and facilities, training professionals who are in contact with children and adolescents (from general health staff to teachers to counsellors) as well as families and/or caregivers, and especially targeted children with autism and mentally handicapped children. Prevention projects predominantly aimed to prevent drug and alcohol abuse, especially targeting vulnerable children and youth, such as street children and orphans. Promotion projects varied considerably, and often aimed to promote well-being, and social and cognitive development. Projects providing psychosocial support received over 70% of total DAMH–CA, and were offered through community–, school– and centre–based initiatives. The project descriptions were quite vague, making it difficult to glean deeper information about the forms of support. Lastly, many of the research studies were epidemiological, and focused on children with autism and special needs.

### Annual trends of DAMH–CA by sector

**Figure 2:**
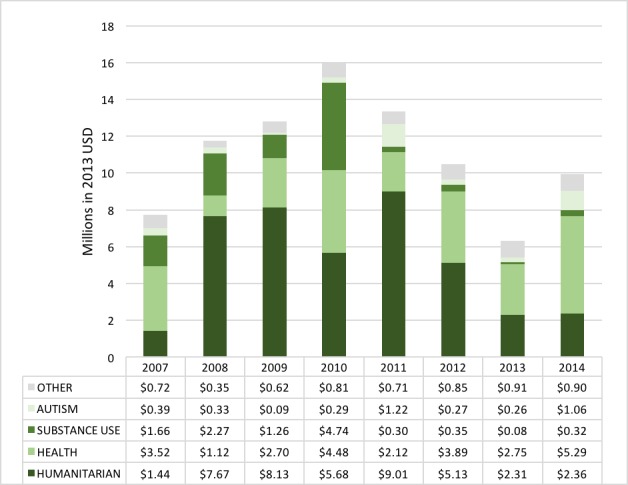
Annual distribution of developmental assistance supporting mental health projects that target children and adolescents (DAMH–CA) by sector, 2007–2014 (US$ millions).

Absolute DAMH–CA in the humanitarian sector decreased 73.8% between 2011 and 2014 (**Figure 2** for annual distribution of DAMH–CA by sector). Since 2012, the health sector received the largest proportion of DAMH–CA, ranging between 37.1% and 53.2%, with an annual average of US$ 3.23 million. DAMH–CA for substance use disorders dropped from 2011, with a 2011–2014 average of just US$ 0.27 million per year, equating to an almost 90% decrease in average spending compared with 2007–2010 (average = US$ 2.48 million). DAMH–CA for autism fluctuated between 2007 and 2014, with an investment of US$ 1.06 million in 2014 (11.0% of DAMH–CA for 2014). Combined annual DAMH–CA for HIV/AIDS, education, rights, and neurological disorders was extremely low in absolute terms, never reaching US$ 1 million (see Table S3 in **Online Supplementary Document(Online Supplementary Document)** for breakdown of annual spending).

### Annual DAMH–CA by project type

**Figure 3:**
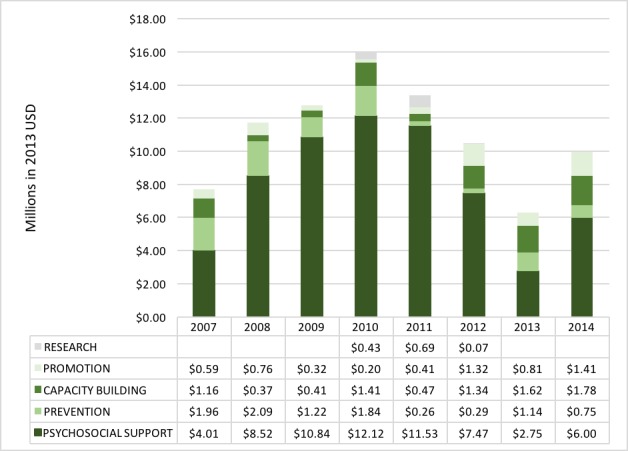
Annual distribution of developmental assistance supporting mental health projects that target children and adolescents (DAMH–CA) by project type, 2007–2014 (US$ millions).

DAMH–CA for projects providing psychosocial support consistently received the largest annual proportions, although decreased in 2014 by more than half compared to its peak in 2010 (**Figure 3**). DAMH–CA for prevention projects more than halved from an average of US$ 1.78 million between 2007 and 2010 to US$ 0.61 million between 2011 and 2014. DAMH–CA for capacity building steadily increased since 2011, although remained low in absolute terms between 2007 and 2014 with an average of US$ 1.7 million per year. Promotion projects consisted of just 6.6% (US$ 5.81 million) of DAMH–CA between 2007 and 2014. Funding supporting research on child and adolescent mental health consisted of just 1.4% of DAMH–CA between 2007 and 2014, and received no funds in 2007–2009, 2013, and 2014.

### Top ten donors and recipients of DAMH–CA, and DAMH–CA per child/adolescent

**Figure 4:**
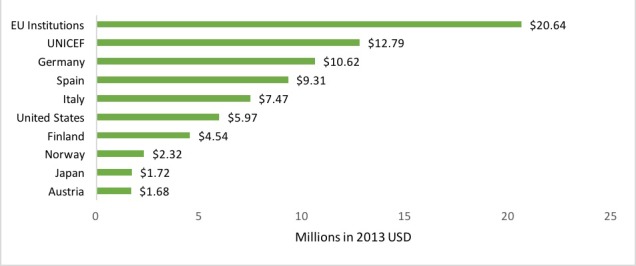
Top ten cumulative donors of developmental assistance supporting mental health projects that target children and adolescents (DAMH–CA), 2007–2014.

Most DAMH–CA was multilateral, ie, aid from international institutions with governmental membership. EU institutions and UNICEF together invested over US$ 33 million – almost 40% – of DAMH–CA between 2007 and 2014 (**Figure 4**). The majority (62.1%) of funding from EU institutions was for humanitarian projects, mainly implemented in the West Bank and Gaza. Many projects by UNICEF supported child friendly spaces, implemented all over the world. Germany provided DAMH–CA for almost one quarter (22.8%) of total DAMH–CA targeting autism, and one fifth of the total DAMH–CA for capacity building. DAMH–CA from Spain constituted almost 60% of total funding for substance use projects, predominantly implemented in South America. DAMH–CA from Italy had no consistent pattern for recipient, project type, or sector. Most aid (77.8%) from the USA was for humanitarian projects in Africa and the Middle East, although several of their projects did not specify the recipient. Over one third (36.4%) of DAMH–CA from Finland, Norway, Japan and Austria was directed Sub–Saharan Africa, predominantly funding psychosocial support projects within the health sector.

**Figure 5:**
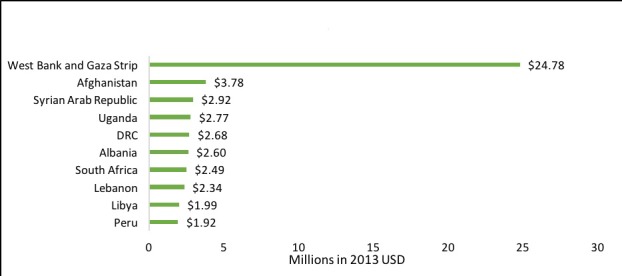
Top ten cumulative recipients of developmental assistance supporting mental health projects that target children and adolescents (DAMH-CA), 2007-2014.

**Figure 6:**
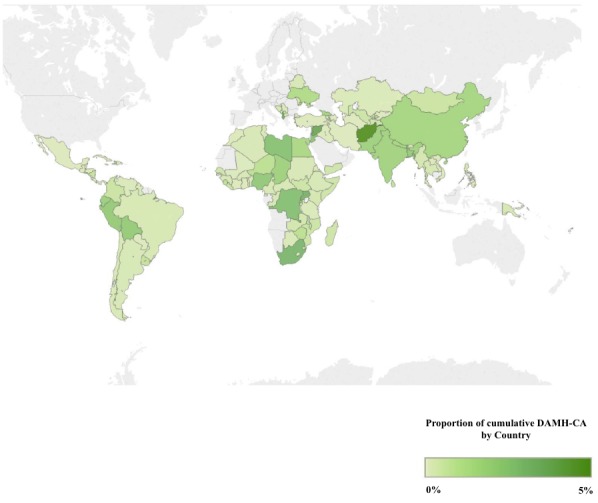
The distribution of developmental assistance supporting mental health projects that target children and adolescents (DAMH–CA) (2007–2014) by recipient country.

The West Bank and Gaza received almost one third of total DAMH–CA (US$ 24.78 million; 28.0%; **Figure 5**), over half of which (US$ 14.4 million) came from EU institutions. All DAMH–CA for Afghanistan was from UNICEF and Germany, and funded psychosocial support and child friendly spaces. Eight out of the 17 projects implemented in Syria were specifically aimed at Iraqi refugees, while the remaining projects encompassed psychosocial support and humanitarian action. Projects in Uganda predominantly provided psychosocial support for vulnerable children, and those in the DRC addressed both the psychosocial impact of conflict on children and capacity building. Most projects in both Albania and Peru were aimed at substance abuse prevention. In South Africa, many projects targeted children affected by HIV/AIDS and other chronic illnesses. Projects in Lebanon often concerned the psychosocial support of refugee children from neighbouring countries. DAMH–CA to Libya consisted of a single ‘emergency response’ disbursement from Italy in 2011. The proportional distribution of cumulative DAMH–CA by country is indicated in **Figure 6**.

Cumulative DAMH–CA (2007–2014) by region was as follows: Asia received US$ 46.24 million (52.3%), Africa received US$ 22.52 million (25.5%), Latin America and the Caribbean (LACAR) received US$ 9.83 million (11.1%), Europe received US$ 4.91 million (5.6%), and Oceania received US$ 0.24 million (0.3%). DAMH–CA for unspecified recipient regions was US$ 4.61 (5.2%). In 2014, the DAMH–CA for one region only (Europe) equated to more than one dollar cent per child/adolescent, three regions (Asia, Africa, Latin America & the Caribbean) received less than one cent per child/adolescent, and Oceania receive no DAMH–CA (as was the case for half of the years between 2007–2014; **Table 2**).

**Table 2:** Annual developmental assistance for DAMH–CA per child/adolescent by region, 2007–2014*

	2007	2008	2009	2010	2011	2012	2013	2014
Africa	<0.01	<0.01	<0.01	<0.01	0.01	<0.01	<0.01	<0.01
Asia	0.02	<0.01	<0.01	<0.01	<0.01	<0.01	<0.01	<0.01
Europe	0.02	0.01	<0.01	0.04	<0.01	<0.01	<0.01	0.02
LACAR	<0.01	<0.01	<0.01	0.02	<0.01	<0.01	<0.01	<0.01
Oceania	0.02	N/I	0.03	0.01	N/I	<0.01	N/I	N/I

DAMH–CA – developmental assistance supporting mental health projects that target children and adolescents, LACAR – Latin America and the Caribbean, N/I – no investment

*Excluded was DAMH–CA for unspecified regions (US$ 4.61; 5.2%).

## DISCUSSION

Analysis of the OECD’s CRS data revealed that DAMH–CA between 2007 and 2014 is low in both absolute terms and relative to DAMH, amounting to a total of US$ 88.35 million (16% of DAMH 2007–2014, annual average = US$ 11.04 million). DAMH–CA steadily increased between 2007 and 2010, progressively decreased from 2010 until 2013, and then increased from the lowest annual total of US$ 6.31 in 2013 to US$ 9.94 in 2014. This pattern could represent a cyclic funding trend, although lack of data on project durations prevent definitive conclusions. Another possible explanation is that funding for children and adolescent mental health is being increasingly channelled into multicomponent development projects which include mental health within their remit; for 2013 and 2014, multicomponent projects constituted 50–70% of total developmental assistance for multicomponent projects combined with DAMH–CA. While integration of mental health services is encouraging, developmental assistance focusing on mental health specifically is still fundamental to build capacity and expertise amongst health practitioners and lay professionals.

704 mental health projects targeting children and adolescents were identified, with an average of US$ 125 500 per project. In 2014, just one region received over US$ 0.01 in DAMH–CA per child/adolescent. Comparison of 2007 and 2014 DAMH–CA per child/adolescent revealed a decrease in two regions and no increase in others, likely due to population increase as well as insufficient funding. These figures indicate that we are still far from the goal of universal mental health care and the minimum investment needed for the scale up of basic mental health care packages [18]. Given that children and young people constitute 44% of the global population, 91% of whom live in LMICs [28], developmental assistance is currently inadequate to support the mental health needs of this demographic. Considering the future ‘human capital’ of this age group and the burden associated with mental illness – 54.2 million YLDs, 6.3 millions YLL, 61.8 million DALYs – low investment in mental well-being constitutes a missed opportunity to mitigate the increasing burden of mental and neurological disorders [4], not to mention a restriction on said generation’s advancement and ability to flourish.

Over 75% of total DAMH–CA was invested in projects implemented within the humanitarian and health sectors, predominantly for the provision of psychosocial support but relatively little in the way of promotion, prevention, or capacity building to sustain a long–term response. A public health approach involving research, prevention, promotion, capacity building and psychosocial support is strongly recommended for mental health, however substance use was the only sector in which there was investment in all project types [29,30]. Autism, as a single disorder, received almost 5% of total DAMH–CA, and encouragingly almost half of this aimed to increase capacity through training professional and lay workers, establishing specialised institutions and services, and supporting parents to respond to their children’s needs. Extremely little funding supports neurological disorders; less than 1% of total DAMH–CA.

Very little DAMH–CA supported mental health projects within the education sector; less than US$ 4 million over the eight years. The relationship between mental health and education is bidirectional, with poor mental health being associated with early school dropout and subsequent long–term negative consequences at the individual, familial, societal and even national level while, in addition to health benefits, psychological well-being has been associated with improved educational outcomes, social relationships and productivity, and reduced absenteeism, anti–social behaviour, and crime [31]. Increased planning and financial collaboration between the health and education sectors is necessary, in particular for programmes aiming to prevent the development of mental disorders and symptoms as these are promoted as an effective and cost–effective approach, and schools are arguably the platform with the highest reach [11–13,32]. Just 11% – under US$ 10 million – of DAMH–CA between 2007 and 2014 funded prevention projects, none of which were based in schools. The only area in which there was substantial investment in prevention programmes was against the development of drug and alcohol use disorders.

Much DAMH–CA from top donors was invested in projects within the humanitarian sector, especially directed at the West Bank and Gaza, which received almost 30% of total DAMH–CA. Many of the top recipient countries are currently or recently affected by armed conflict, or neighbouring those affected by conflict. There is a distinct lack of investment trends, suggesting a lack of direction for children and adolescent mental health care. To improve this, and increase autonomous and sustainable mental health care in LMICs, increased investment is required especially in research and capacity building. Cumulative DAMH–CA for these project types amounted to just US$ 1.20 (1.4%) and US$ 8.57 million (9.7%), respectively. Investment in research and capacity building is essential to equip countries with the necessary data to guide their financing of mental health services, and strengthen the design, implementation, evaluation, and scale–up of evidence–based interventions.

It must be noted that the figures presented here are an underestimate of mental health funding for children and adolescents, as they do not include governmental and private organisation funds. This is a limitation of the study, especially regarding the estimate for research funding, as much of this funding comes from such sources. However, a database (or similar) detailing research projects funded through different agencies is not available, and therefore including research projects only funded by specific funding bodies would have clouded the conceptual clarity of the study. It was also not possible to estimate the proportion of DAMH–CA for multicomponent and community–targeted projects due to their heterogeneity, although the project descriptions suggested that mental health and children and adolescents, respectively, were not the primary targets of these projects. Increased reporting transparency and detail in the CRS would give clearer insight into the distribution of the benefits of these projects. The exclusion of multicomponent programmes is the likely reason for discrepancies with previous DAMH estimates, as well as the inclusion of additional keywords geared towards the psychosocial side of mental illness, such as ‘well-being’ and ‘therapy’. Compared to previous methodologies (eg, [23]), the method used here to identify projects targeting children and adolescents was more thorough, as each project was considered for inclusion or exclusion as opposed to the calculation of developmental assistance based on population proportions.

Given the widespread consensus that the optimal method for negotiating the increasing burden of mental health is through targeting children and adolescents as part of a life–course approach [4,13], increased DAMH–CA is crucial. Projects aiming to promote well-being and prevent mental disorders merit a substantial increase in investment, as they have been demonstrated to be both effective and cost–effective, particularly those implemented in schools and through national policy [32]. Research and capacity building especially require increased funding, to ensure appropriate and sustainable care and promotion of mental health and well-being of future generations.
