# Supplementary material for: Developmental assistance for child and adolescent mental health in low– and middle–income countries (2007–2014): Annual trends and allocation by sector, project type, donors and recipients
Source: J Glob Health. 2017 Dec 19;7(2):020901. doi: 10.7189/07.020901 (PMC5737098; doi:10.7189/07.020901)
Supplement: Online Supplementary Document [file jogh-07-020901-s001.pdf]

Online Supplementary Document

Turner et al. Developmental assistance for child and adolescent mental health in low- and middle-income countries (2007-2014): Annual trends and allocation by sector, project type, donors and recipients

J Glob Health 2017;7:020901

Table S1. Keywords used to search for mental health projects in the Creditor Reporting System, 2007-2014

English	Italian	French	Portuguese	Spanish	German	Dutch
Abuse<br>(drug/<br>substance)	Abuso (di droghe/ sostanze)	Abus<br>(de substance/ de drogue)	abuso (de drogas/ substâncias)	abuso (de drogas/ substancias)	Drogenmissbrauch	misbruik<br>drugsmisbruik<br>middelenmisbruik
Alcohol	Alcol/alcool	Alcool	álcool	alcohol	alkohol	alcohol
Cognit-<br>(as in, cognitive, cognition)	Cognizione	Cognit-<br>cognitif<br>cognitive	cognit- Cognitivo	cognitiv-<br>cognición	kognitiv	cognitieve<br>kennis
Counsel-<br>(as in counsellors, counselling)	Consulen-<br>(Consulente/<br>consulenza)	thérapeute	terapeuta	terapeuta	Therapeut<br>Therapeutin	raadgever<br>begeleiding
Distress	Angoscia	Détresse<br>Angoisse	aflição	Angustia<br>afficción	Not / Leid / Leiden / Bedrängnis	nood/angst

Mental health<br>Mental illness<br>mental disorder<br>mental disability<br>mental distress	salute mentale<br>disordine mentale<br>disordini mentali<br>malattia/e mentale/i<br>disturbi mentali<br>disturbo mentale<br>ritardo mentale<br>disabilità mentale<br>handicap mentale<br>mentalmente	Santé mentale<br>Maladie mentale<br>(MALADES MENTAUX)<br>Désordre mental<br>Maladies mentales<br>Trouble mentale<br>Troubles mentaux<br>Déficience mentale<br>Handicap mental<br>Incapacité mentale<br>Handicaps mentaux<br>Déficiences mentales<br>Mentalement	saúde mental<br>doença mental (doenças mentais)<br>distúrbio mental<br>mentalmente<br>doentes mentais<br>doente mental<br>mentalemente doente<br>problemas mentais<br>perturbações mentais<br>deficiência mental<br>incapacidade mental	salud mental<br>enfermedad mental<br>trastorno mental<br>discapacidad mental<br>incapacidad mental<br>enfermedades mentales<br>deficiencia mental<br>trastornos mentales	psychische / mentale / seelische Gesundheit<br>Psychische Erkrankung<br>Psychische / mentale Störung<br>Psychische Gesundheit<br>Geisteskrankheit<br>Geistiger Zurückgebliebenheit<br>Geistiger Behinderung<br>Geistesstörung<br>Psychischen Störungen<br>Geisteskrankheiten	geestelijke gezondheid (mental health)<br>psychische stoornis/aandoening (mental disorder[s])<br>Geestelijke stoornis<br>Geestesziekte<br>verstandelijke handicap<br>Geestelijke handicap<br>Intellectuele handicap<br>mentale handicap<br>geestelijk gehandicapt<br>geestesziekte

					Geistig<br><br>psychisch	geestelijk
Psych-	Psic-	Psych-	psic-	psico<br>psíquica	Psych-	Psych-
Therap- (therapy, therapeutic)	Terap-	Thérap-	Terap-	Terapia<br>terapéutica	Therapie therapeutisch	therapie<br>therapeutische
Trauma (trauma, traumatised, traumatized)<br>PTSD	Trauma<br>DPTS<br>Disturbo post traumatico da stress	Trauma-<br>SPTD	trauma - traumatismo, traumatizado	trastorno de estrés postraumático<br>(TEPT)	Trama / Seelischer Schock (traumatisiert)<br><br>Posttraumatische Belastungsstörung	trauma<br>PTSS (PTSD)
Well being, well-being, wellbeing	Benessere	Bien-être	bem-estar	bienestar	Wohlbefinden	welzijn (well-being)
Addiction	dipendenza	addiction	Vício<br><br>Dependência habituação	Adicción	Sucht / Abhängigkeit	verslaving (addiction)
Antidep-	Antidep	antidépres	anti-dep	antidepresivo	antidep	antidepress- (antidepressant[s])
Anxiety	Ansia	anxiété	ansiedade	Ansiedad	Angst / Angstgefühl	angst

						ongerustheid bezorgdheid
Autism	Autismo	autisme	autismo	autismo	Autismus	autisme
Bipolar	Bipolare (disturbo)	bipolaire	bipolar	bipolar	bipolar	bipolaire
Depress-	Depress-	dépress-	depressão	depresión	depress-	depressie
Dyslexia	Dislessia	dysléxie	dislexia	dislexia	Legasthenie	dyslexie
Mood	Umore	Humeur	humor	humor	Laune / Stimmung	stemming
Schizo-	Schizo-	schizo-	esquizo-	esquizofrenia	Schizo	schizo-
Self harm<br>Suicide	Suicidio<br>Autolesionismo	mutilation<br>suicide	auto-mutiação, suicidio	Suicidio<br>autolesiones	Selbstbeschädgung / Selbstverletzung<br>Suizid / Freitod / Selbstmord	zelf pijniging<br>zelfmoord
ADHD	ADHD	TDAH	ADHD	TDAH	ADHD	ADHD
Neuro-	neuro	neuro	neuro	neuro	neuro	Neuro-

Table S2. Definitions of sector and project type codes

	Code	Definition
**Sector**	Education	All school-based programmes
	Health	Projects addressing health or social welfare
	Humanitarian	Including conflict, emergency and natural disaster
	Rights	Projects addressing human and legal rights, including the right to education and healthcare

	Substance use	Including alcohol- and drug-related disorders
	HIV/AIDS	Projects addressing children and adolescents with HIV/AIDS, or orphaned due to the disease
	Neuro	Neurological disorders, including epilepsy
	Autism	All projects pertaining to autism, coded as a sector due to the high investment it received as a single disorder
**Project type**	Research	Projects entailing all forms of research into children and adolescent mental health (including prevalence, causes, treatment efficacy etc.)
	Capacity building	Projects aiming to increase and/or improve health systems and services, training, or partnership formation between organisations or sectors
	Prevention	Projects aiming to prevent and/or protect against the development of mental disorders, noted to frequently target vulnerable populations, e.g. street children
	Promotion	Including advocacy, policy, awareness-raising and wellbeing promotion
	Psychosocial support	Including support, care, counselling, interventions, rehabilitation, treatment and social inclusion

Table S3. Breakdown annual DAMH-CA for HIV/AIDS, rights, neuro, and education (USD millions)

	2007	2008	2009	2010	2011	2012	2013	2014	Grand Total (USD millions)
**HIV/AIDS**	$0.59	$0.14	$0.13	$0.37	N/I	$0.02	N/I	N/I	$1.26
**Rights**	N/I	$0.05	$0.27	N/I	$0.21	$0.02	N/I	$0.13	$0.69
**Neuro**	N/I	N/I	N/I	$0.03	$0.07	$0.08	$0.14	$0.21	$0.53
**Education**	$0.12	$0.16	$0.21	$0.41	$0.43	$0.73	$0.77	$0.56	$3.39

Text S1. Coding process

Codes for sectors were based on the CRS categorisation (health, education, humanitarian) and inductive categorisation from the project descriptions/keywords (rights, HIV/AIDS, neuro, autism). Project types were inductively coded based on the project descriptions.

For cases where projects belonged to more than one sector, e.g. ‘humanitarian’ and ‘education’, a form of hierarchy was decided on. All projects carried out in humanitarian settings were coded ‘humanitarian’ (even if they were school-based, for example), while all other school-based projects were ‘education’. The ‘Sector name’ project information was also used to inform categorisation.

Text S2. Calculating DAMH-CA by region and per child/adolescent

Population figures were taken from a United Nations Population Division (dataset ‘Population by broad age groups- both sexes’) [33], where data on region, age group, and gender was available. The population data was for 2005-2015, and not available by year. The following steps were taken to calculate annual DAMH-CA per child/adolescent by region:

Step 1 Population figures for each region for the child/adolescent age group (0-19 yrs) were recorded, and then high income country populations for the same age group were subtracted for each region.

$$\text{Population}_{\text{Region/Age/Year}} - \text{Population}_{\text{HICs/Age/Year}}$$

Step 2a To calculate the annual rate of child/adolescent population change (i.e. increase or decrease) by region, the 2005 population figure was subtracted from the 2015 population figure. Then this figure was divided by 10 (i.e. 2015-2005; the number of years) to estimate the average annual rate of population change, for each region.

$$(\text{Population}_{\text{Region/Age/2015}} - \text{Population}_{\text{Region/Age/2005}}) / 10$$

Step 2b To calculate the child/adolescent annual population for each region, first the average annual rate of population change for each region was multiplied by the number of years between the year of calculation and 2005, denoted X (for example 2009, would be calculated at 2009-2005=4, hence X=4). Then this calculated figure was added to the population figure of 2005 (per region, by age).

$$\text{Population}_{\text{Region/Age/2005}} + \{[(\text{Population}_{\text{Region/Age/2015}} - \text{Population}_{\text{Region/Age/2005}}) / 10]*X\}$$

Step 3 DAMH-CA was divided by the population estimates for each region by year, to give annual DAMH-CA per child/adolescent

$$\text{DAMH-CA}_{\text{Region}} / \text{Population}_{\text{Region/Age/Year}}$$
